# Non-Invasive Characterization of Single-, Double- and Triple-Viral Diseases of Wheat With a Hand-Held Raman Spectrometer

**DOI:** 10.3389/fpls.2020.01300

**Published:** 2020-09-03

**Authors:** Charles Farber, Rebecca Bryan, Li Paetzold, Charles Rush, Dmitry Kurouski

**Affiliations:** ^1^Department of Biochemistry and Biophysics, Texas A&M University, College Station, TX, United States; ^2^Department of Plant Pathology, Texas A&M AgriLife Research and Extension Center at Amarillo, Amarillo, TX, United States; ^3^The Institute for Quantum Science and Engineering, Texas A&M University, College Station, TX, United States

**Keywords:** coinfection, disease detection, Raman spectroscopy, *Triticum aestivum*, virus, wheat

## Abstract

Plant diseases can reduce crop yield by up to 100%. Therefore, timely and confirmatory diagnosis of plant diseases is strongly desired. Typical pathogen assaying methods include polymerase chain reaction (PCR) and enzyme-linked immunosorbent assay (ELISA). These approaches are quite useful but are also time-consuming and destructive to the sample. Raman spectroscopy (RS) is a modern analytical technique that enables non-invasive plant disease detection. In this study, we report on Raman-based detection of wheat diseases caused by wheat streak mosaic virus (WSMV) and barley yellow dwarf virus (BYDV). Our results show that RS can be used to differentiate between healthy wheat and wheat infected by these two viruses. We also show that RS can be used to identify whether wheat is infected by these individual viruses or by a combination of WSMV and BYDV, as well as WSMV, BYDV, and Triticum mosaic virus (TriMV). We found that wheat spectra showed non-linear spectroscopic responses to coinfection by different viruses. These results suggest that RS can be used to probe pathogen-specific changes in plant metabolism. The portable nature of this approach opens the possibility of RS directly in the field for confirmatory diagnostics of viral diseases.

## Introduction

Wheat (*Triticum aestivum*) is one of the widely most grown cereal crops in the world: in 2017 alone, over 750 million tons of wheat were produced across 124 different countries (FAOSTAT Compared Data). Consumption of wheat accounts for over 18% of the total food supply of the world (FAOSTAT Compared Data). Thus, improvement and protection of this crop is critical for long-term food security (Food and Agriculture Organization of the United Nations, 2009). As with most plants, wheat is susceptible to a wide variety of pathogens, including bacteria, viruses, and fungi, all of which can greatly impact yield. Among all these pathogens, viruses are often challenging to detect, as their visual symptoms may resemble those of nutrient deficiency (Freije et al., 2016). Additionally, plants are often subject to coinfection by different types of viruses simultaneously, leading to symptoms that might not resemble those of any single virus alone (Mascia and Gallitelli, 2016). Although numerous viruses can infect wheat, three of them cause the most devastating crop losses: wheat streak mosaic virus (WSMV), Triticum mosaic virus (TriMV), and barley yellow dwarf virus (BYDV) (Hadi et al., 2011; Kaddachi et al., 2014).

WSMV is a filamentous ssRNA virus of the *Potyviridae* family and vectored by the wheat curl mite (WCM) *Aceria tosichella*. Both the virus and its vector can thrive on alternative hosts, such as volunteer wheat or other grasses growing near the primary wheat crop, allowing these to survive between wheat growing seasons. The virus causes chlorotic streaks running parallel to the veins of the leaves, ultimately leading to leaf necrosis and plant death. WSMV is often found infecting its host alongside TriMV, a recently discovered virus of wheat localized to the Great Plains region of the United States (Seifers et al., 2008). Like WSMV, it is a filamentous ssRNA member of the *Potyviridae* and is vectored by WCM (Seifers et al., 2009). Alone, TriMV symptoms resemble those of WSMV. If these two viruses are present together, WSMV is almost always found at a higher titer in infected leaf tissue and disease symptoms are amplified (Bryan et al., 2019). BYDV is a spherical ssRNA virus of the *Luteoviridae*. It is a widespread, economically important virus, vectored by aphids instead of WCM. BYDV also causes chlorosis and mottling of infected plant leaves. BYDV-infected plants exhibit purpling of leaf tips and have severe plant stunting (Miller and Rasochová, 1997). BYDV has been found to coinfect wheat together with WSMV and TriMV.

Confirmatory diagnostics of viral disease can be achieved using polymerase chain reaction (PCR) or quantitative (q)PCR. Although accurate, both PCR and qPCR are time and labor consuming, destructive to the sample, and require a complicated primer design to detect all infectious agents present in the sample (Martinelli et al., 2014). Therefore, a quick, non-invasive, portable, and confirmatory approach for diagnostics of viral disease is strongly desired. We have previously shown that Raman spectroscopy (RS) can be used to detect and identify plant diseases caused by fungi, viruses, and bacteria (Farber and Kurouski, 2018; Farber et al., 2019; Sanchez et al., 2020). This is highly important for agriculture because RS is non-invasive, non-destructive, and typically requires little-to-no sample preparation. Specifically, we employed a portable Raman spectrometer and were able to detect and identify four different fungal corn kernels (Farber and Kurouski, 2018). Coupling RS to chemometrics, we showed that the accuracy of such diagnostics of these fungal diseases was 100%. We also demonstrated that RS can be used for pre-symptomatic detection of rose rosette disease (RRD) on roses. RRD is caused by a virus that is vectored by mites (Farber et al., 2019). We found that the leaves of infected asymptomatic plants exhibited higher content of phenylpropanoids and molecules with carboxylic moieties compared to leaves of healthy roses. These findings suggested that Raman diagnostics is based on detection and identification of pathogen-induced changes in host molecules. However, one may question whether such changes are virus specific. To answer this question, Mandrile and colleagues investigated the use of RS for differentiation between viruses of tomato (Mandrile et al., 2019). Their findings suggest that RS can be used to differentiate between two different viruses. However, it is unclear whether RS can differentiate between viruses and their coinfecting combinations. To answer this question, we collected Raman spectra from wheat plants infected by WSMV, BYDV, WSMV and BYDV, WSMV and TriMV, as well as WSMV, BYDV and TriMV. These spectra were compared to the spectra collected from healthy wheat plants.

## Materials and Methods

### Vector Cultivation and Inoculation

WSMV colonies were maintained on Karl 92 wheat. New wheat, at the 2-leaf stage and Feeke’s scale 2–3, was infested using a snip of wheat containing at least 20 wheat curl mites from an established plant.

TriMV colonies were maintained on RonL wheat, but otherwise treated the same as the WSMV.

BYDV was not cultivated and was only found in naturally-infected field-grown wheat plants.

### Wheat

Naturally infected wheat was grown at the USDA Conservation and Production Research Laboratory in Bushland, TX. These fields were planted to hard red winter wheat cv. Karl 92 in late September, at a seed rate of 31 kg/acre. Urea was applied preplant at a rate of 45 kg/acre, and Ally herbicide was applied pre-plant at 28 g/acre. Fields were irrigated as needed during the season with a center pivot sprinkler system. Wheat was harvested in late June with a John Deere 9500 combine mid- to late July. Samples 8–44 were grown in the field.

Positive controls for WSMV and TriMV (Samples 1–7) infected wheat came from greenhouse grown plants used to maintain the colonies described previously. Because these plants are used to maintain the vectors, no information about the infection stage of the samples was maintained.

Healthy control plants were grown in the same greenhouse in a Bugdorm to keep insects out.

A representative leaf of each infection status can be found in **Figure S1**.

#### PCR/qPCR

##### Nucleic acid extraction

Total RNA was extracted from wheat leaf tissue using the Qiagen RNeasy Plant Mini Kit (Qiagen, Germantown, MD) per manufacturer’s instructions.

##### Real-time PCR analysis of wheat – Disease Status

One-step Fast Absolute Quantification tests were conducted using an Applied Biosystems ViiA 7 Real-Time PCR System. PCR amplification was done using a TaqMan Fast Virus 1-step Master Mix kit (Applied Biosystems, Austin, TX.)

For the WSMV test, each reaction mix contained 1x Taqman Fast Virus 1-step Master Mix (Applied Biosystems), 0.4 µM forward primer Wsm F 9075, 0.4µM reverse primer Wsm R 9196, and 0.25µM Wsm P 9142 FAM-Probe (Tatineni et al., 2010). For the TriMV and BYDV tests, reaction mix concentrations were the same as for the WSMV test. The TriMV test used forward primer Tri F 9754, reverse primer Tri R 9875, and Trip 9802 FAM-Probe (Tatineni et al., 2010). The BYDV test used a 1X primer/probe combination BYDV Primers Combined FAM/NFQ-MGB Probe (Applied Biosystems, Pleasanton, CA). The Real-time PCR thermal cycling profile was the program: 50°C for 5 min, 95°C for 20 s followed by 40 cycles of denaturing at 95°C for 3 s then annealing at 60°C for 30 s.

### Copy Number Determination

Copy number was determined by conducting a Standard Curve Absolute Quantification test. This test requires cDNAs derived from the viral RNAs. Reverse transcription was first performed using Qiagen’s Omiscript RT kit and prescribed manufacturer’s protocol.

For the WSMV test, each reaction mix contained 1x Taqman Fast Advanced Master Mix (Applied Biosystems), 0.3 µM forward primer *Wsm* F 9075, 0.3µM reverse primer *Wsm* R 9196, and 0.25µM *Wsm* P 9142 FAM-Probe (Tatineni et al., 2010). 20 ng of template cDNA was added to each reaction for all three virus assays. For the TriMV test, reaction mix concentrations were the same as for the WSMV test. Forward primer Tri F 9754, reverse primer Tri R 9875, and Trip 9802 FAM-Probe (Tatineni et al., 2010) were used. The BYDV primers and probe were designed in our lab with the Thermofisher Custom Taqman^®^ Assay Design Tool, using sequence accession number EF521836-1: forward primer BYDV F2910, sequence 5’GAAGGAGGCGCCGTAGAA 3’, reverse primer, BYDV R2949, sequence 5’CCTGCTCGATTGGGTTGGA 3’ and probe, BYDV2928, sequence FAM 5’ACCACTGGCCGAACTG 3’ MGB-NFQ. The BYDV primers and probe can be requested from ABI, Assay ID APXGRYP, 20x Custom Taqman^®^ Gene Expression Assay BYDV and used 1x per reaction. The Real-time PCR thermal cycling profile was the default program: 95°C for 20 s followed by 40 cycles of denaturing at 95°C for 1 s then annealing at 60°C for 20 s.

WSMV, TriMV, and BYDV virus copy numbers in leaf samples were determined using plasmid DNA standards. Plasmid DNA for all three viruses were synthesized by Invitrogen (Thermo Fisher, Pleasanton, CA). A 300 bp WSMV insert was synthesized from accession number NC001886 at position 9001-9300. A 300bp TriMV insert was synthesized from accession number FJ669487 at position 9661-9960. A 300bp BYDV insert was synthesized from accession number EF521836-1 at position 2801-3100. The vector was the commercial pMA-T plasmid. The plasmid size was 2674 bp. Sequence identity within the insertion sites was 100% as checked by Invitrogen. Approximately 5 µg plasmid DNA was synthesized. The website “DNA Copy Number and Dilution Calculator” (Thermo-Fisher) was used to calculate copy number dilutions. A stock 0.5 ng/µl dilution was made with RNAse free water, then standard serial dilutions of 10^7^, 10^6^, 10^5^ 10^4^, 10^3^, 10^2^, and 10 copies/µl were made. Standard curve quantification real-time tests were run on prepared samples.

The results of virus quantification are reported in **Table S1**. A threshold copy number of 17 was set to identify samples as being positive for a viral infection. We observed substantial variation in copy number across our samples. In some cases, we had below 1000 copies of WSMV (samples 26–32), whereas others showed over 1 billion copies (samples 15 and 18). For the purposes of this proof-of-concept manuscript, we only accounted for the binary positive-or-negative assignment in assigning our spectra to categories for the PLS-DA and ANOVA analyses. Further studies should explore how copy number influences the Raman spectra.

#### Raman Spectroscopy

qPCR-tested samples analyzed with Raman spectroscopy included those that were: positive for all three viruses, positive for either WSMV or BYDV only, positive for WSMV and TriMV only, positive for WSMV and BYDV only, and negative for all three viruses. Samples positive for TriMV were also always positive for WSMV, so no samples positive for TriMV only or TriMV and BYDV only were evaluated.

Wheat leaves were scanned in a laboratory using an 830 nm Agilent Resolve spectrometer in the surface scanning mode with the following parameters: power = 320 mW, acquisition time = 1 second and spectral resolution = 15 cm^-1^. 5-10 spectra were acquired per leaf, but varying sample quality and availability resulted in fewer usable spectra. Number of leaves analyzed and spectra used per infection status are summarized in **Table 1**. Spectra were automatically baseline corrected by the instrument software then averaged together for use in the figures.

**Table 1:** Summary of samples analyzed by Raman spectroscopy for this study.

Health Status (qPCR-confirmed)	Number of Leaves sampled	Spectra used
Healthy (H)	4	20
WSMV (W)	6	36
BYDV (B)	5	24
WSMV + TriMV (WT)	2	16
WSMV + BYDV (WB)	22	134
WSMV + BYDV + TriMV (WBT)	2	16

#### Statistical Analysis

Spectra were assigned classes based on their qPCR-confirmed combination of infecting viruses (or lack thereof) and imported into the MATLAB addon PLS_Toolbox ver. 8.7 (Eigenvector Research, Inc.) Spectra were preprocessed (see *Results* for further details) and used to build a partial least squares discriminant analysis (PLS-DA) model. This model was found to be significant at the α=0.05 level following 100 iterations of the permutation test (Van Der Voet, 1994; Thomas, 2003).

Differences in normalized intensity were evaluated by Analysis of Variance (ANOVA) in MATLAB. Spectra were first normalized to the 1440 cm^-1^ band, then the anova1 function was used to conduct the ANOVA analyses at the specific bands of interest. Significant differences were then identified using the multcompare function, which uses a Tukey-Kramer multiple comparison test to determine which pairs are significantly different following an ANOVA. Reported differences are significant at the α=0.05 level. Confidence intervals (**Figure S2**) and tables (**Tables S2**-**S15**) associated with these ANOVAs can be found in the supporting information.

## Results and Discussion

**Figure 1:**
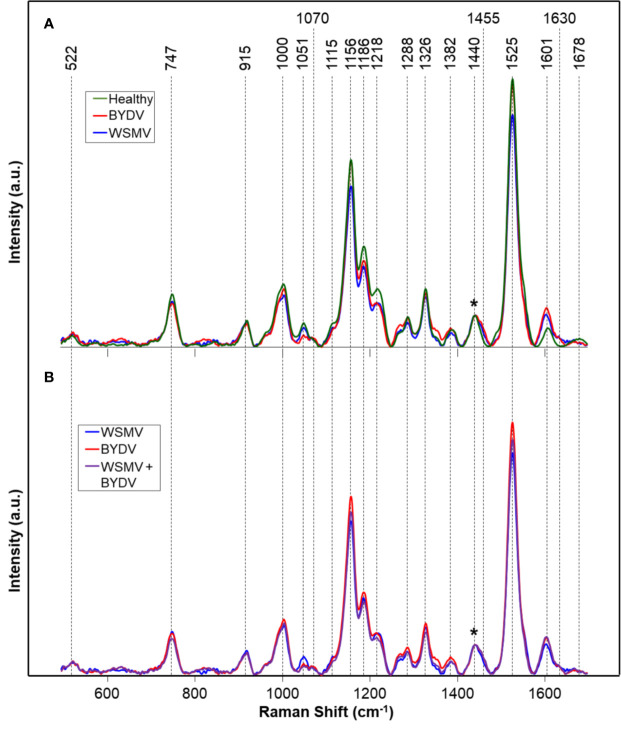
Raman spectra of: **(A)** Healthy and WSMV- or BYDV- infected wheat leaves and **(B)** the combination of these two viruses. Normalization band at 1440 cm^-1^ is marked by an asterisk (*). A.u., Arbitrary units.

We analyzed spectra collected from leaves of wheat plants infected with WSMV (W) and BYDV (B), a combination of WSMV and BYDV (WB), a combination of WSMV and TriMV (WT), and finally WSMV, BYDV, and TriMV (WBT) simultaneously. In the spectra of wheat leaves, we found vibrational bands that can be assigned to carbohydrates, carotenoids, phenylpropanoids, and proteins (**Figure 1**, **Table 2**). We also observed aliphatic (CH_2_ and CH_3_) vibrational bands that cannot have unambiguous assignment to specific compounds due to their abundance in all classes of biological molecules. Because of this, we decided to normalize our data to the 1440 cm^-1^ band for visual comparison of spectra.

**Table 2:** Vibrational band assignments for wheat leaf spectra.

Band (cm^-1^)	Vibrational mode	Assignment
522 (vw)	ν(C-O-C) glycosidic	cellulose (Edwards et al., 1997)
747 (w)	γ(C–O-H) of COOH	pectin (Synytsya et al., 2003)
915 (w)	ν(C-O-C) in plane, symmetric	cellulose, lignin (Edwards et al., 1997)
1000 (m)	in-plane CH_3_ rocking of polyene	carotenoids (Adar, 2017; Devitt et al., 2018)
1051 (w)	ν(C-O)+ν(C-C)+δ(C-O-H)	cellulose, phenylpropanoids (Edwards et al., 1997)
1070 (vw)	ν(CO) of secondary alcohol	cellulose (Edwards et al., 1997)
1115 (w)	sym ν(C-O-C), C-O-H bending	cellulose (Edwards et al., 1997)
1156 (s)	asym ν(C-C) ring breathing	carotenoids (Jehlička et al., 2014), cellulose (Edwards et al., 1997)
1186 (m)	ν(C-O-H) next to aromatic ring+σ(CH)	xylan (Mary et al., 2012; Agarwal, 2014)
1218 (m)	δ(C-C-H)	aliphatics (Yu et al., 2007), xylan (Agarwal, 2014)
1288 (w)	δ(C-C-H)	aliphatics (Yu et al., 2007)
1326 (w)	δCH_2_ bending vibration	cellulose, phenylpropanoids (Edwards et al., 1997)
1382 (w)	δCH_2_ bending vibration	aliphatics (Yu et al., 2007)
1440 (w)	δ(CH_2_)+δ(CH_3_)	aliphatics (Yu et al., 2007)
1455 (w)	δCH_2_ bending vibration	aliphatics (Yu et al., 2007)
1525 (vs)	-C=C- (in plane)	carotenoids (Adar, 2017; Devitt et al., 2018)
1601 (w)	ν(C-C) aromatic ring+σ(CH)	phenylpropanoids (Agarwal, 2006; Kang et al., 2016)
1630 (vw)	C=C-C (ring)	phenylpropanoids (Agarwal, 2006; Kang et al., 2016; Pompeu et al., 2017)
1678 (vw)	C=O stretching, amide I	proteins (Devitt et al., 2018), carbonyl-containing compounds

vw, very weak; w, weak; m, medium; s, strong; vs, very strong.

Vibrational bands at 522, 747, 915, 1051, 1070, 1115, 1186, and 1218 cm^-1^ can be assigned to carbohydrates. These molecules have scaffold properties (cellulose and xylan), as well as serve as energy storage (pectin and other carbohydrates). These bands are primarily associated with the vibrations of C-O-C, C-O, C-O-H, and C-C-H in rings (Edwards et al., 1997). Specific vibrational band assignments can be found in **Table 2**. The bands at 1525 and 1000 cm^-1^ can be assigned to the in-plane polyene rocking of CH_3_’s and C=C in-plane vibration of carotenoids, common pigment molecules in plants (Adar, 2017; Devitt et al., 2018). The vibrational band at 1156 cm^-1^ can be potentially assigned to both carotenoids and carbohydrates. This band can generally be assigned to C-C ring breathing, which occurs in both molecules. The presence of stronger 1525 and 1000 cm^-1^ bands alongside this one suggests that this band may be associated with carotenoids. However, further chemical analyses, such as mass spectrometry, are required to determine the exact identity of the compounds contributing to this band.

Phenylpropanoids are a broad class of phenolic compound which includes lignin and suberin. These molecules have a variety of functions, from structural integrity to pathogen resistance.(Vogt, 2010) The vibrational bands at 1600 and 1630 cm^-1^ can be assigned to the aromatic ring and C=C-C of ring vibrations in phenylpropanoids (Agarwal, 2006; Kang et al., 2016; Pompeu et al., 2017). The band at 1326 cm^-1^, associated with CH_2_ vibrations, could potentially be assigned to carbohydrates or phenylpropanoids. Proteins in Raman spectra are typically identified by the presence of a vibrational band at about 1678 cm^-1^, known as the Amide I band (Devitt et al., 2018). This band is also observed in many carbonyl-containing compounds, such as the phenylpropanoids. Further analysis would be required to unambiguously assign this band to a specific molecule. The vibrational bands we observe in the spectra collected from wheat leaves are in good agreement with previously reported Raman spectra of leaves (Yeturu et al., 2016; Farber et al., 2019; Sanchez et al., 2019).

### Comparison of Healthy and Infected Wheat Spectra

We sought to determine whether RS coupled to statistical methods could be used to differentiate wheat plants infected with different combinations of viruses. We first employed partial least squares discriminant analysis (PLS-DA), a supervised classification method, to distinguish between healthy, W and B-infected plants. We built a model, Healthy-Single-Single (HSS), using 58 spectra from the healthy, B and W datasets. Spectra were truncated to the range of 500–1700 cm^-1^, area-normalized to the 1440 cm^-1^, then interval partial least squares (iPLS) was used to select the 228 wavenumbers that resulted in the highest cross-validation performance. HSS, containing nine latent variables (LVs), was found to be on average 90% accurate in differentiating among healthy and single infection wheat as shown in the cross-validation confusion table (**Table 3**). The model explained 99.4, 0.27, 0.07% of the variation in the first three latent variables, respectively. This suggests that RS in combination with chemometrics can enable a highly accurate prediction of wheat disease status in single virus scenarios.

**Table 3:** Confusion table for cross-validation of the HSS model.

	Members	Correct	WSMV+	BYDV+	Healthy
WSMV+	36	88%	14	1	3
BYDV+	24	91%	0	22	1
Healthy	20	95%	0	1	32

Correct – true positive rate of assigning a spectrum to its correct category.

Next, to identify potential markers of disease, we conducted ANOVAs on the intensities at bands associated with the metabolites described above. First, we searched for bands that could uniquely identify healthy wheat. We found that the bands at 747 and 1218 cm^-1^ were significantly more intense in the healthy wheat spectra than spectra of any infected type. Additionally, we identified several additional bands, 1186, 1326, 1455, 1601, 1630, and 1678 cm^-1^, where the healthy spectra show overlap with only one type of infection. These sets of Raman bands could potentially be useful markers to identify healthy plants among diseased ones.

We then sought to determine whether Raman markers exist for specific disease combinations in terms of WSMV (shortened to W). We found that while W could not be identified uniquely from any ANOVA, bands at 747, 1051, 1218, 1455 cm^-1^ showed a significant difference between the W infection and one of its coinfections. The two double coinfections, WT and WB, could be distinguished from each other by almost all bands analyzed, including 747, 1000, 1051, 1186, 1218, 1288, 1326, 1455, 1601, and 1630 cm^-1^. In all except 1455, 1601 and 1633 cm^-1^, WT is significantly more intense than WB. Finally, the triple infection, WBT, could be uniquely identified by the 1186 cm^-1^ band and shows overlap with only one other combination at 1218 cm^-1^. These results indicate that RS can identify biochemical markers of different WSMV-associated wheat diseases.

BYDV (shortened to B) alone, like W, could not be uniquely identified using ANOVA. However, at 1051 cm^-1^, B, WB and WBT clearly separated from healthy, W and WT, suggesting this band could be a generic marker of infections that include B. Additionally, the bands at 1455, 1601, and 1630 show significant differences between the B combinations and WT. While no band enables differentiation of B and WB, the single infection could be differentiated from WBT by bands at 1156, 1186, 1218, and 1525 cm^-1^. Using this large number of marker bands across different infections, RS can be used to enable differentiation of WSMV, BYDV, and TriMV-associated wheat diseases.

While these observed differences could be used for disease diagnostics, they are also indicative of biochemical changes under different infecting conditions. We observed that in general, WT was significantly more intense than WB. As described previously, TriMV is known to enhance the symptoms of WSMV. Our results suggest that perhaps BYDV weakens the symptoms of WSMV or suppresses them with its own symptoms. The fact that we do not observe WSMV coinfections changing in tandem as is seen in BYDV coinfections (1051 cm^-1^) provides further evidence that BYDV may alter the biochemical responses elicited by WSMV. Additionally, we found that across most bands examined with ANOVA, WBT showed the lowest intensity. This may indicate that the combination of all three viruses places greater strain on the host metabolism, lowering all relevant metabolite concentrations. The only bands that do not show this trend are 1601 and 1630 cm^-1^, the phenylpropanoid bands. The buildup of the compound in the infected system is reasonable, as it is associated with cell wall hardening. These observations show the potential of RS for examining coinfecting pathogens on a pathogen-to-pathogen level.

Through RS, we identified marker bands that are associated with major players in the infection cycle. 1186 and 1218 cm^-1^ are associated with xylan, a major cell wall component in grasses such as wheat. Changes observed in this band may suggest strengthening or weakening of the cell wall. We observed that this band was more intense in the spectra of healthy plants than all infected classes except B, though WT was very close. This observation suggests that the cell wall (through xylan degradation) is weakened in the presence of many of these viral infections. Additionally, we found that in the spectra collected from WBT-infected wheat, these bands were less intense than the corresponding bands in all other classes (WT, WB…), suggesting that triple infection may put a greater burden on xylan maintenance than other combinations of viruses.

## Conclusions

We have demonstrated the potential of RS as a tool for the detection and identification of individual and combinatorial viral infections. Using RS, we could not only distinguish healthy plants from those with single-virus infections, but also between those single infections and their combination, suggesting that combinatorial viral infections are more than the sum of their parts. Our PLSDA model had 90% accuracy for differentiation of healthy and single-infection wheat. Additionally, using ANOVA, we described Raman bands that could potentially be used to diagnose wheat viral diseases down to the combination level and described their biochemical relevance. Together, our results suggest that RS may be a powerful alternative or supplement to PCR for the detection and analysis of wheat viruses.

## Data Availability Statement

The raw data supporting the conclusions of this article will be made available by the authors, without undue reservation.

## Author Contributions

CF: Investigation, Data curation, methodology. RB: Investigation, data curation. LP: Investigation CR: Funding acquisition, supervision. DK: Methodology, funding acquisition, supervision.

## Funding

Support for this project was provided by a grant from the Texas A&M AgriLife Research Insect Vector initiative to CR and the Governor’s University Research Initiative grant to DK (no. 12-2016, M1700437).

## Conflict of Interest

The authors declare that the research was conducted in the absence of any commercial or financial relationships that could be construed as a potential conflict of interest.
