# Supplementary material for: Non-Invasive Characterization of Single-, Double- and Triple-Viral Diseases of Wheat With a Hand-Held Raman Spectrometer
Source: Front Plant Sci. 2020 Sep 3;11:01300. doi: 10.3389/fpls.2020.01300 (PMC7495046; doi:10.3389/fpls.2020.01300)

Non-invasive Characterization of Single-, Double- and Triple-Viral Diseases of Wheat with a Hand-held Raman Spectrometer

Supplementary Material


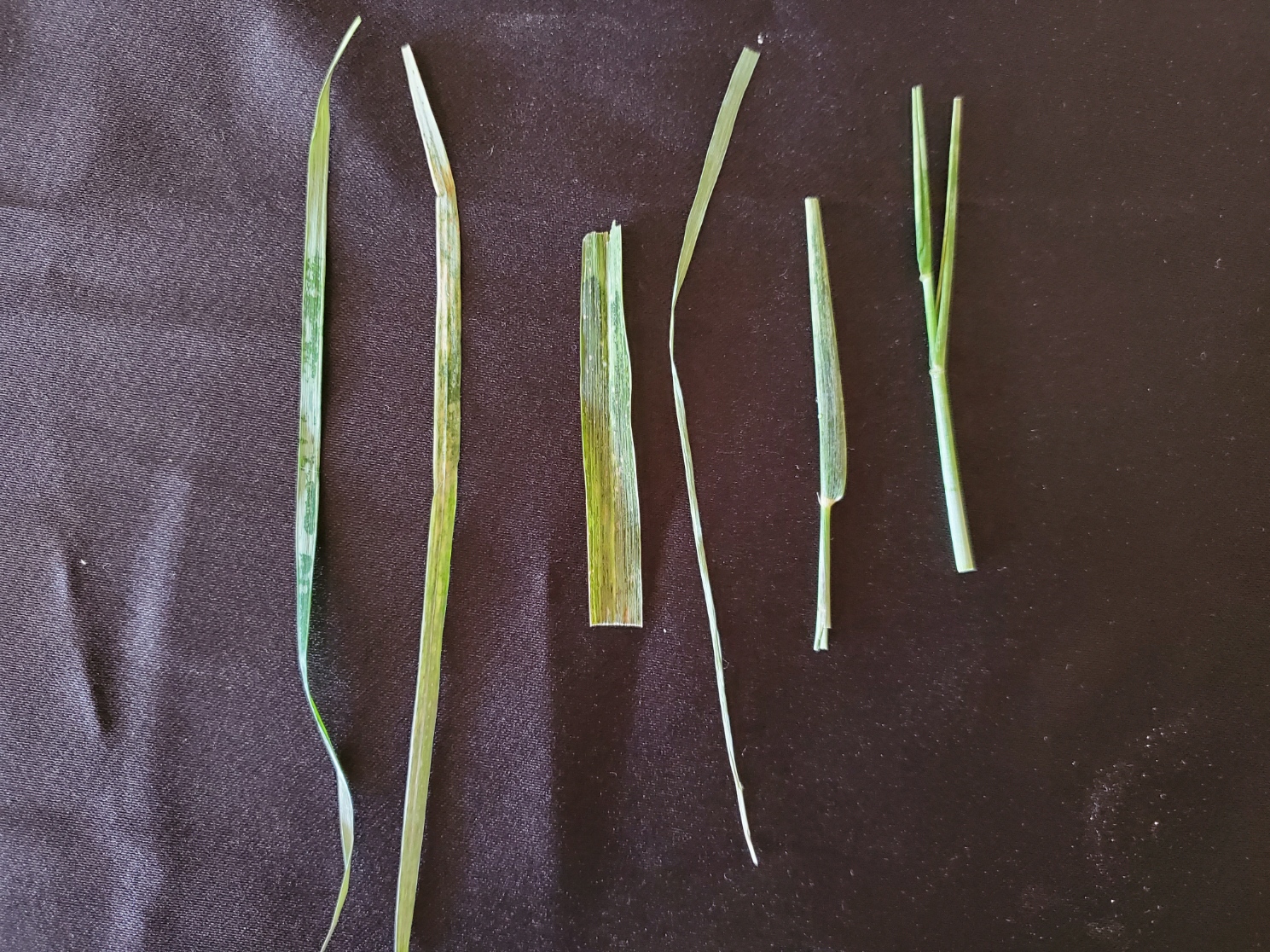


Figure S1. Photograph of example wheat leaves scanned in the study. From left to right: healthy (sample 39), WSMV (sample 1), BYDV (sample 12), WSMV + TriMV (sample 4), WSMV + BYDV (sample 9), WSMV + BYDV + TriMV (sample 8).

Figure S2. 95% confidence intervals for the true mean normalized intensity of Raman spectra acquired from wheat leaves. Non-overlapping intervals indicate that the two groups are significantly different.

Table S1. qPCR copy numbers and assigned statuses for all plants analyzed in the study.


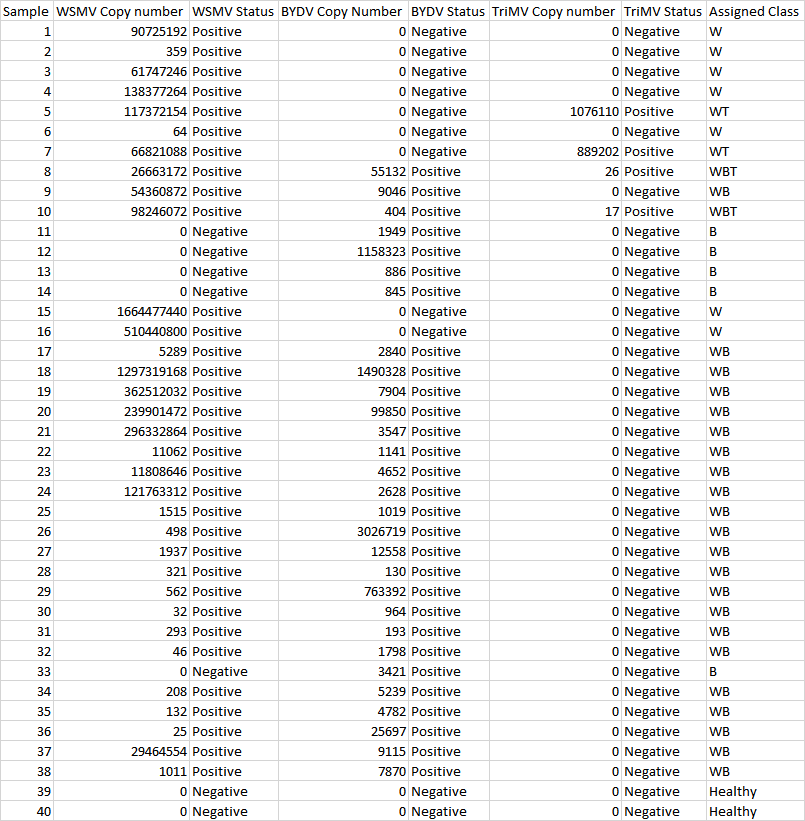


Tables S2-14. ANOVA tables for all ANOVAs conducted in this study. SS – sum of squares; df – degrees of freedom; MS – Mean squared error; F – F-statistic. P-value < 0.05 indicates one group is significantly different from another.

Table S15. Summary table of Tukey-Kramer multiple comparison test p-values for all ANOVAs conducted in this study. P-value < 0.05 indicates the groups are significantly different.


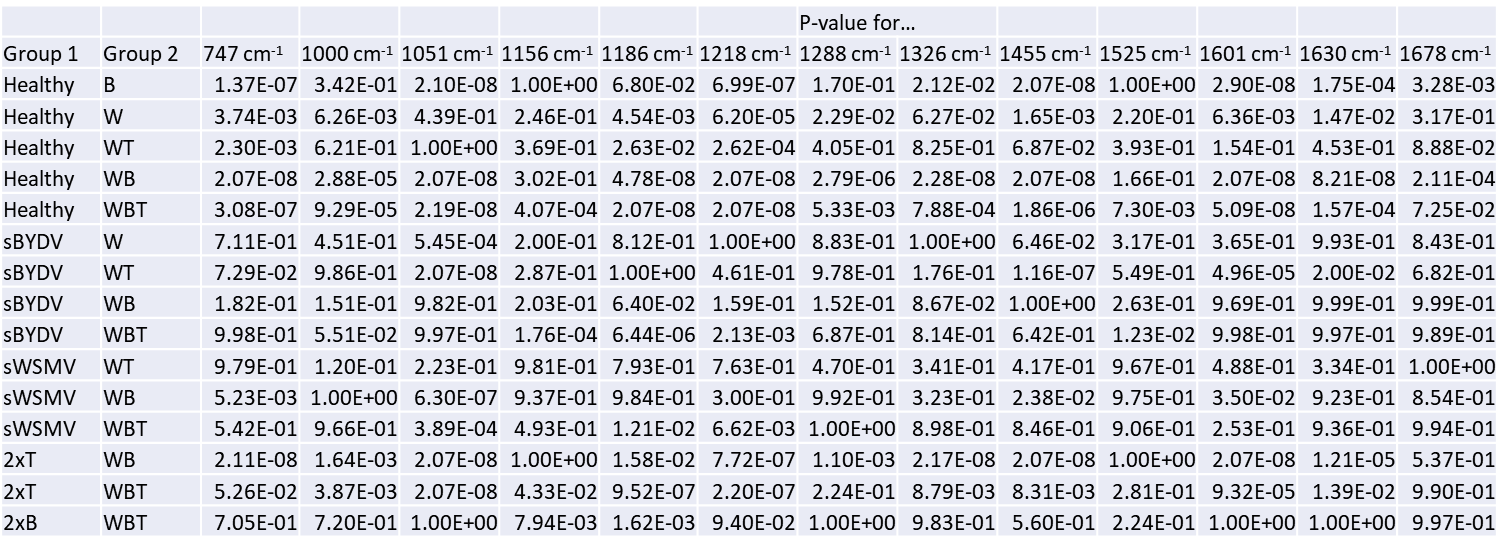

Supplement: Supplementary file 1 [file DataSheet_1.docx]
